# The effects of laparoscopic Roux-en-Y gastric bypass and one-anastomosis gastric bypass on glycemic control and remission of type 2 diabetes mellitus: study protocol for a multi-center randomized controlled trial (the DIABAR-trial)

**DOI:** 10.1186/s13063-022-06762-3

**Published:** 2022-10-22

**Authors:** A. van Rijswijk, N. van Olst, A. S. Meijnikman, Y. I. Z. Acherman, S. C. Bruin, A. W. van de Laar, C. C. van Olden, O. Aydin, H. Borger, U. H. W. Beuers, H. Herrema, J. Verheij, J. A. Apers, F. Bäckhed, V. E. A. Gerdes, M. Nieuwdorp, L. M. de Brauw

**Affiliations:** 1 Department of Surgery, Spaarne Gasthuis, Spaarnepoort 1, 2134 TM Hoofddorp, the Netherlands; 2 Department of Vascular Medicine, Amsterdam UMC, Meibergdreef 9, 1105 AZ Amsterdam, the Netherlands; 3 Department of Internal Medicine, Spaarne Gasthuis, Spaarnepoort 1, 2134 TM Hoofddorp, the Netherlands; 4 Department of Gastroenterology and Hepatology and Tytgat Institute for Liver and Intestinal Research, Amsterdam UMC, Meibergdreef 9, 1105 AZ Amsterdam, the Netherlands; 5 Department of Pathology, Amsterdam UMC, Meibergdreef 9, 1105 AZ Amsterdam, the Netherlands; 6 Department of Surgery, Franciscus Gasthuis, Kleiweg 500, 3045 PM Rotterdam, the Netherlands; 7 Wallenberg Laboratory, Department of Molecular and Clinical Medicine, University of Gothenburg, SE-413 45 Gothenburg, Sweden; 8 Novo Nordisk Foundation Center for Basic Metabolic Research, University of Copenhagen, Blegdamsvej 3B, Building 7, DK-2200 Copenhagen, Denmark; 9 Department of Internal Medicine, Amsterdam UMC, Meibergdreef 9, 1105 AZ Amsterdam, the Netherlands; 10 Institute for Cardiovascular research, Amsterdam UMC, Meibergdreef 9, 1105 AZ Amsterdam, the Netherlands

**Keywords:** One-anastomosis gastric bypass, Roux-en-Y gastric bypass, Randomized controlled trial, Type 2 diabetes mellitus, Metabolic outcome

## Abstract

**Background:**

Metabolic surgery induces rapid remission of type 2 diabetes mellitus (T2DM). There is a paucity of high level evidence comparing the efficacy of the laparoscopic Roux-en-Y gastric bypass (RYGB) and the laparoscopic one-anastomosis gastric bypass (OAGB) in glycemic control. Also, the mechanisms that drive the conversion of T2DM in severe obese subjects to euglycemia are poorly understood.

**Methods:**

The DIABAR-trial is an open, multi-center, randomized controlled clinical trial with 10 years follow-up which will be performed in 220 severely obese patients, diagnosed with T2DM and treated with glucose-lowering agents. Patients will be randomized in a 1:1 ratio to undergo RYGB or OAGB. The primary outcome is glycemic control at 12 months follow-up. Secondary outcome measures are diverse and include weight loss, surgical complications, psychologic status and quality of life, dietary behavior, gastrointestinal symptoms, repetitive bloodwork to identify changes over time, glucose tolerance and insulin sensitivity as measured by mixed meal tests, remission of T2DM, presence of non-alcoholic fatty liver disease/non-alcoholic steatohepatitis in liver biopsy, oral and fecal microbiome, cardiovascular performance, composition of bile acids, and the tendency to develop gallstones.

**Discussion:**

The DIABAR-trial is one of the few randomized controlled trials primarily aimed to evaluate the glycemic response after the RYGB and OAGB in severe obese patients diagnosed with T2DM. Secondary aims of the trial are to contribute to a deeper understanding of the mechanisms that drive the remission of T2DM in severe obese patients by identification of microbial, immunological, and metabolic markers for metabolic response and to compare complications and side effects of RYGB and OAGB.

**Trial registration:**

ClinicalTrials.gov NCT03330756; date first registered: October 13, 2017.

**Supplementary Information:**

The online version contains supplementary material available at 10.1186/s13063-022-06762-3.

## Background

Metabolic surgery has proven to be a successful long-term solution in the treatment of severe obesity and its comorbidities. It induces rapid remission of type 2 diabetes mellitus (T2DM) and is more effective than intense medical treatment [1–6]. Although the incidence of (severe) obesity and T2DM are on the rise, it is undecided which metabolic procedure has the most optimal risk-to-benefit ratio in terms of the effect on T2DM, post-operative complications and side-effects [3]. Some favor the Roux-en-Y gastric bypass (RYGB) over the sleeve gastrectomy in patients with T2DM, but despite its good results, the RYGB is considered an advanced and complex procedure [7]. The one-anastomosis gastric bypass (OAGB) has been introduced in 2001 as a novel and simplified variant of the RYGB. Available single-arm studies report favorable results of OAGB in the treatment of T2DM and, although little evidence is available comparing the efficacy of RYGB and OAGB in the treatment of T2DM, some suggest a benefit from OAGB over RYGB [8–14]. To the best of our knowledge, the DIABAR-trial is one of the few randomized controlled trials with primary endpoint to evaluate the glycemic response after laparoscopic RYGB and OAGB in patients diagnosed with severe obesity and T2DM, with up to 10 years of follow-up. The DIABAR-trial will clarify the benefit of these two procedures in the treatment of T2DM and directly compare complications and side effects, which will aid patients and physicians in an evidence-based decision upon the most optimal metabolic procedure. The secondary aim is to phenotype the patients enrolled in the trial and to identify driving mechanisms in the interplay between obesity and metabolic disease by identification of microbial, immunological and metabolic markers for metabolic response.

## Methods/design

### Objective of the study

The primary objective of the study is to compare glycemic control 12 months after RYGB and OAGB. Secondary aims of the study are to evaluate remission of T2DM (up to 10 years follow-up), weight loss, surgical complications, psychologic status and quality of life, dietary behavior, gastrointestinal symptoms, to phenotype patients and to identify driving mechanisms in the interplay between obesity and metabolic disease. To this end, extensive data collection will be performed. This data collection includes the assessment of liver biopsies obtained during surgery upon the presence of non-alcoholic fatty liver disease (NAFLD)/non-alcoholic steatohepatitis (NASH), oral and fecal microbiome, cardiovascular performance, glucose tolerance and insulin sensitivity as measured by mixed meal tests (MMTs), repetitive bloodwork to identify changes over time, biopsies of adipose tissue and jejunum during surgery, the composition of bile acids, and the tendency to develop gallstones. This data will be used to identify microbial, immunological and metabolic markers for metabolic response.

### Study design and sites

The DIABAR-trial is a two-armed, multi-center, open randomized controlled clinical trial comparing the effect of the RYGB (control) and the OAGB (intervention) on glycemic control. Participating centers are the Spaarne Gashuis (Hoofddorp, the Netherlands), MC Slotervaart (Amsterdam, the Netherlands), and the Franciscus Gasthuis (Rotterdam, the Netherlands). The DIABAR-trial started in October 2017 at MC Slotervaart (Amsterdam, the Netherlands) and the trial continued in March 2019 at the Spaarne Gasthuis (Hoofddorp, the Netherlands) by the same surgical staff and research team. In August 2020, the Franciscus Gasthuis (Rotterdam, the Netherlands) was added as a participating center. Materials will be stored in the DIABAR-biobank, which is located in the affiliated Amsterdam University Medical Center (AUMC) (Amsterdam, the Netherlands). Study materials will be processed in the laboratory of the AUMC and in the Wallenberg laboratory (University of Gothenburg, Gothenburg, Sweden).

**Fig. 1:**
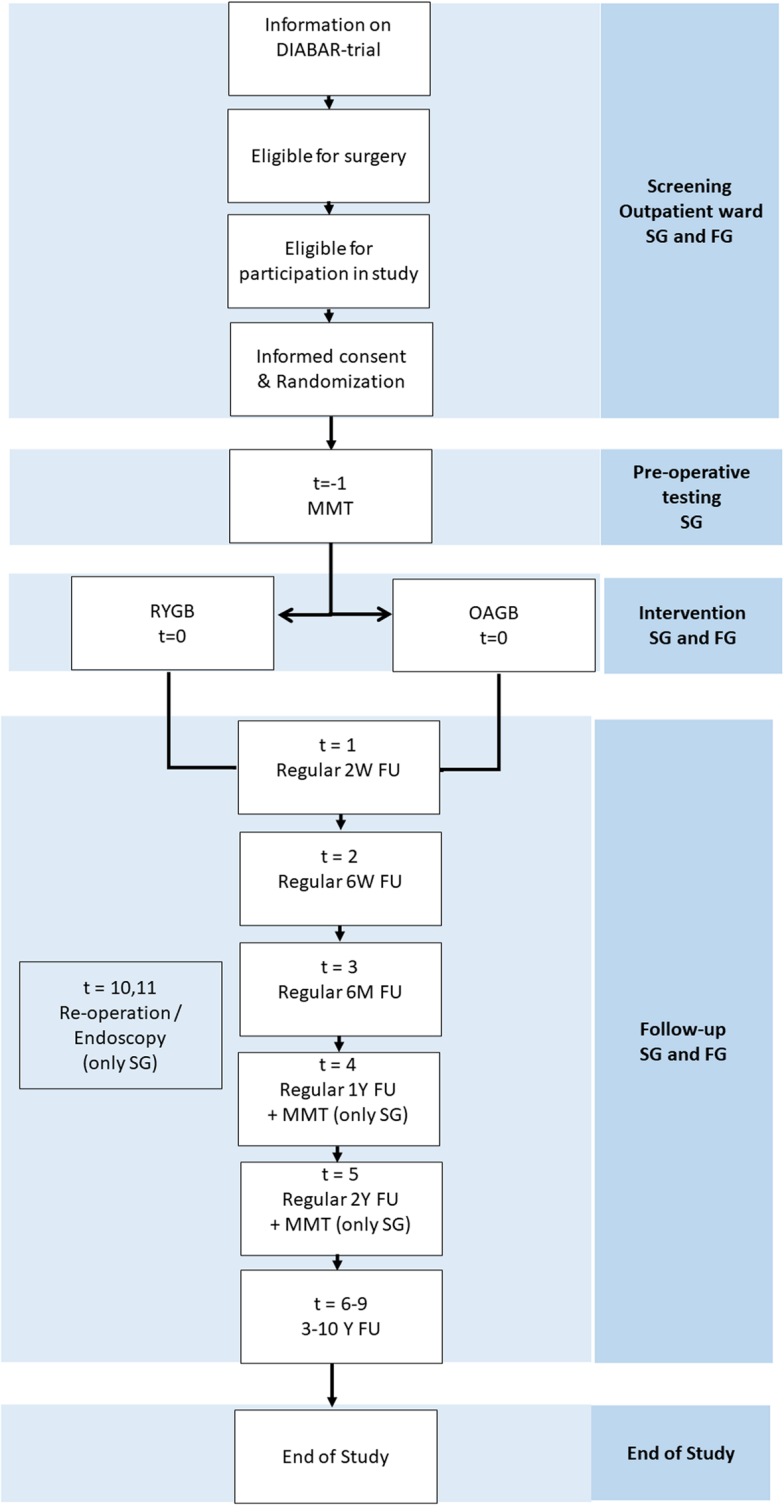
Outline of study enrolment, randomization, intervention and follow-up. *FG* Franciscus Gasthuis, *FU* follow-up, *MMT* mixed meal test, *M* month, *OAGB* one-anastomosis gastric bypass, *RYGB* Roux-en-Y gastric bypass, *SG* Spaarne Gasthuis,* t* time, *W* week

The DIABAR-trial is closely related to the BARIA-study, a longitudinal cohort study in 1500 patients with severe obesity. In the BARIA study, systems biology is used to identify microbial, immunological, and metabolic markers for metabolic response prior to and after bariatric surgery [15]. The BARIA-study is conducted at the Spaarne Gasthuis and the existing infrastructure of the BARIA-study can be used in the DIABAR-trial. This explains why patients participating from the Spaarne Gasthuis will be asked to partake in a more extensive study protocol than patients enrolling from the Franciscus Gasthuis (Table 1). In Fig. 1 an outline of study enrolment, randomization, intervention and follow-up is shown.

**Table 1:** Study procedures in the Spaarne Gasthuis and the Franciscus Gasthuis

		Enrolment allocation	Post-allocation								
Timepoint		***t***=-2	***t*** = -1	***t*** = 0	***t*** = 1	***t*** = 2	***t*** = 3	***t*** = 4	***t*** = 5	***t***=6-9	***t***=10
	Medical center	Screening	< 42 days of surgery	Day of surgery	14 D	42 D	6 M	1 Y	2 Y	3,4,5,10 Y	Re-operation^**a**^/endoscopy
**Enrolment/allocation**											
Eligibility screen	SG/FG	x									
Informed consent and allocation	SG/FG	x									
**Demographics**											
Medical history	SG/FG	Reg	x				x	x	x	x	
Medication check	SG/FG	Reg	x	x	x	x	x	x	x	x	x
**Biometrics**											
Physical examination	SG/FG	x	x	x		x	x	x	x	x	
CO/SVR/BS	SG		x					x	x		
**Blood**											
Laboratory work	SG/FG	Reg		Reg			Reg	Reg	Reg	Reg	Reg
MMT	SG		x					x	x		
Extra blood samples	SG		x	x				x	x	x	x
**Questionnaires**											
Psychological questionnaires	SG/FG		x				x	x	x	x	
GIQLI	SG/FG		x					x	x	x	
Dietary and satiety list	SG		x					x	x		
**Oral, fecal, and urine samples**											
Urine sample	SG				x	x	x			x	
Feces sample	SG				x	x	x			x	
24-h feces and urine	SG			x				x	x		
Oral microbiome	SG		x					x	x	x	
**Surgery and tissue samples**											
RYGB or OAGB	SG/FG			x							
Small intestine tissue sample	SG			x							x^a^
Subcutaneous adipose tissue	SG			x				x	x		x
Visceral adipose tissue	SG			x							x
Omental tissue	SG			x							x
Liver biopsy	SG			x						x	x
Portal blood sample	SG			x^a^							x^a^
Bile acids and bile salts	SG			x^a^							x^a^
Surgical endpoints	SG/FG				x	x	x	x	x	x	
**Other**											
Ultrasound gallbladder	SG		x^a^					x^a^	x^a^		

*BS* baroreflex sensitivity, *MMT* mixed meal test, *D* days, *CO* cardiac output, *GIQLI* gastro-intestinal quality of life, *FG* Franciscus Gasthuis, *FU* follow-up, *M* months, *OAGB* one-anastomosis gastric bypass, *SG* Spaarne Gasthuis, *SVR* systemic vascular resistance, *RYGB* Roux-en-Y gastric bypass, *Reg* regular, *t* time, *Y* year

^a^If applicable

### Study population

The study population will consist of 220 severely obese subjects diagnosed with and treated for T2DM who are scheduled for bariatric surgery.

### Inclusion criteria

Male and female patients, aged between 18 and 65 years and with a BMI of at least 35 kg/m^2^ at intake and 50 kg/m^2^ or less on the day of surgery, are eligible for participation in the study. Only patients with proven T2DM, Hba1c > 7.0% at diagnosis, and treated with glucose-lowering agents at intake at the bariatric outpatient clinic or at least 2 months prior to surgery, and with an American Society of Anesthesiologist Classification (ASA) of three or less will be enrolled in the study.

### Exclusion criteria

Patients will be excluded on the following basis:

- Known genetic basis for insulin resistance or glucose intolerance
- Type 1 diabetes mellitus
- Prior bariatric surgery
- Auto-immune gastritis
- Gastro-esophageal reflux disease (confirmed by endoscopy or the use of a proton-pump inhibitor indicated by complaints of gastro-esophageal reflux)
- Known presence of a large hiatal hernia requiring concomitant surgical repair
- Coagulation disorders or a hemoglobinopathy
- Uncontrolled hypertension (RR > 150/95 mmHg)
- Renal insufficiency (creatinine > 150 μmol/L)
- Pregnancy or breastfeeding
- Alcohol or drug dependency
- Participation in any other therapeutic study that may influence primary or secondary endpoints
- Patients who are considered incapable to fully understand the study and implications of participation in the study (e.g., as a consequence of a language barrier, psychiatric disease or mental disabilities) as judged by the coordinating researcher or the surgeon

### Informed consent and randomization

Patients are first informed on the study during the preoperative screening program, which consists of consultations with a surgeon, an internist, a dietician, a psychologist, and meetings for patient education. If patients proceed towards surgery, they are screened for inclusion into the study. Patients are asked for written informed consent to participate in the study by the surgeon and also to store study materials in the DIABAR-biobank. After informed consent is obtained by the surgeon at the outpatient clinic, patients will be randomly assigned by the surgeon to undergo a RYGB or OAGB in a 1:1 ratio. Randomization will be performed with the use of electronic block-randomization with the program *Castor Electronic Data Capture* (Ciwit BV, the Netherlands). Block size varies between four and six; there is no stratification. Patients and surgeons are directly informed on the outcome of the randomization.

### Treatment

All patients receive pre-, peri-, and post-operative care according to the local bariatric protocol. All patients are urged to lose bodyweight before surgery to decrease surgical risks. Patients are not required to undergo a low calorie liquid diet prior to surgery. Thrombosis prophylaxis is administered in the first post-operative week and a proton-pump inhibitor is prescribed for 3 months after surgery. Patients are advised to lifetime use of vitamins. Initial diabetes management after surgery is coordinated by the internist and during the first months after surgery transferred to a patient’s own diabetes team.

### RYGB

**Fig. 2:**
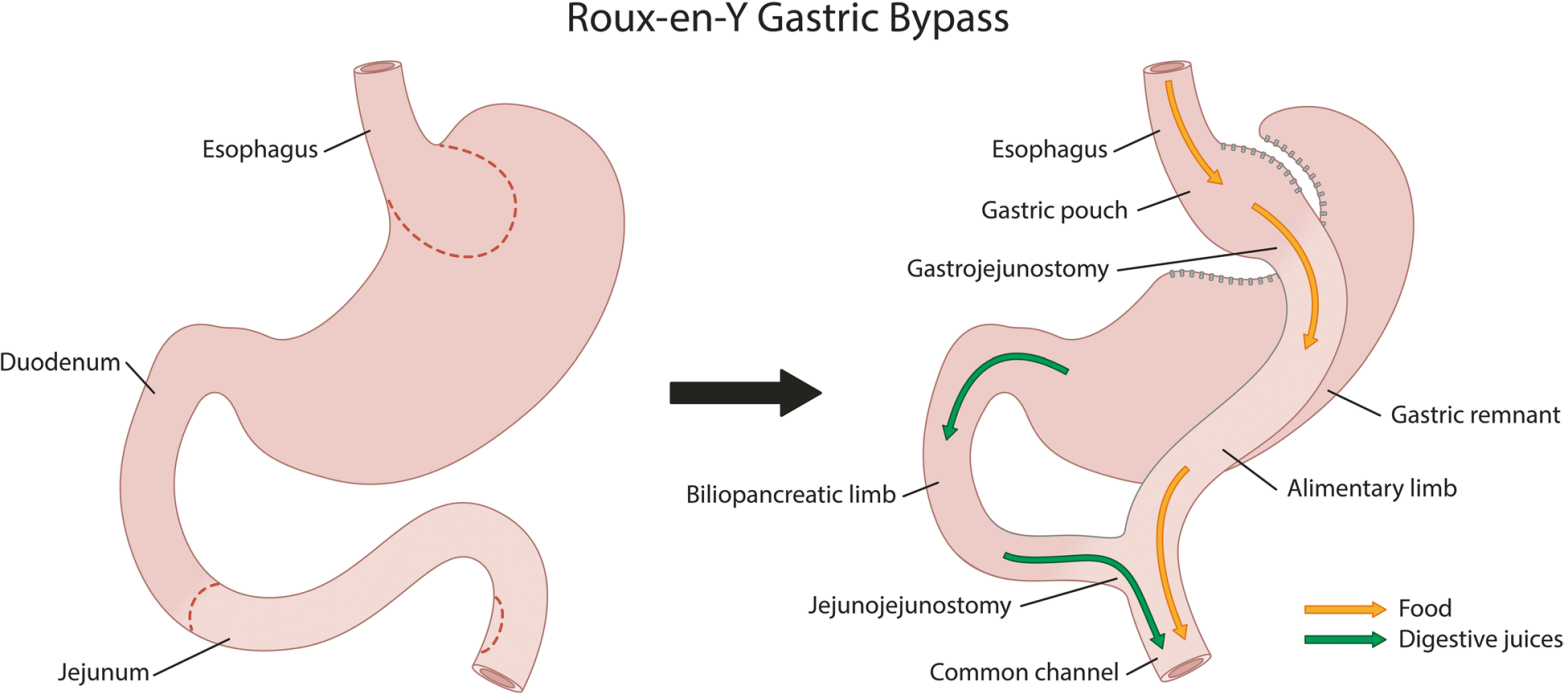
Roux-en-Y gastric bypass

A RYGB consists of a gastric pouch with a volume of approximately 25 ml, a 50-cm biliary limb, and a 150-cm alimentary limb. The alimentary limb is brought up antecolic and antegastric and is anastomosed to the gastric pouch with a linear stapler and a running barbed wire. The jejunojejunostomy connects the alimentary and the biliary limb into a common channel. This anastomosis is fully stapled or closed with a running barbed wire [16, 17]. The mesenteric windows are closed with staples. The RYGB is depicted in Fig. 2.

### OAGB

**Fig. 3:**
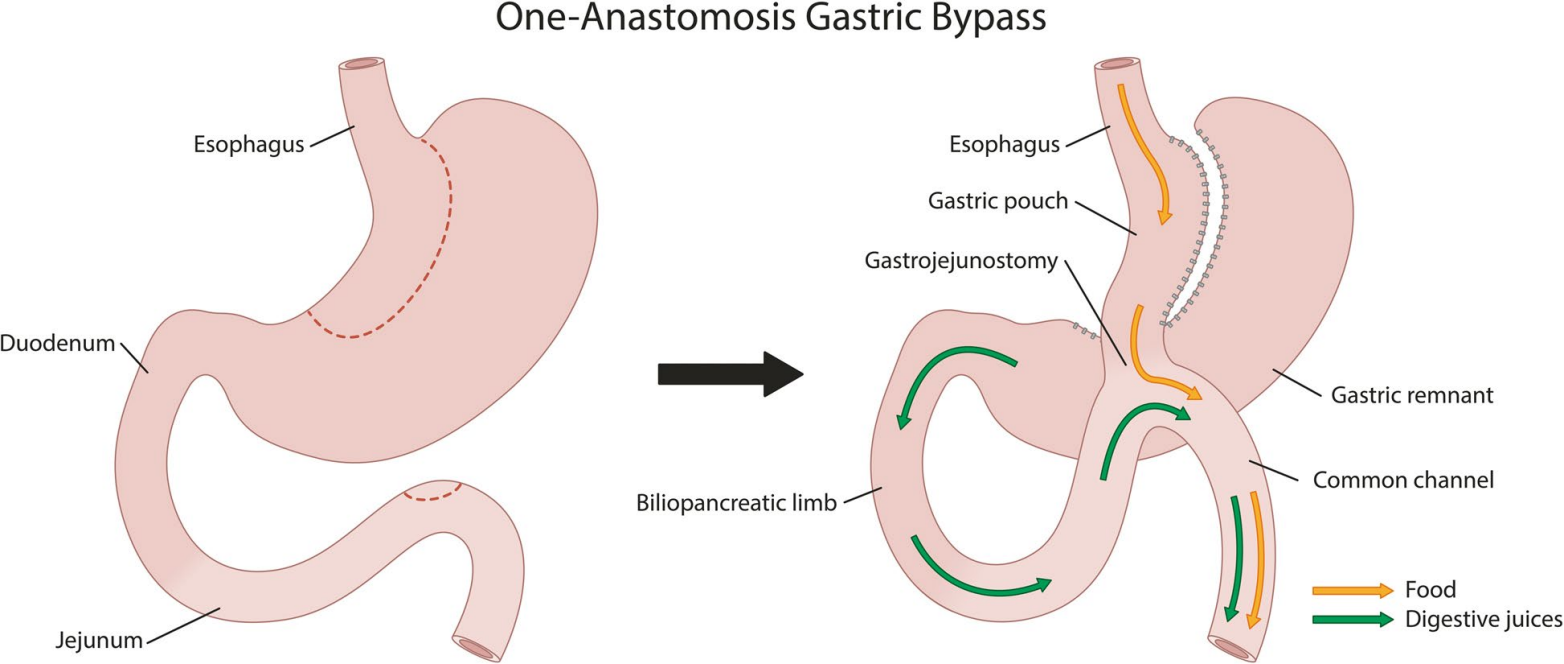
One-anastomosis gastric bypass

The OAGB consists of a long-sleeved and narrow stomach pouch parallel to the lesser curvature, from the Crow’s foot up to the angle of His. The pouch is connected to the jejunum 200 cm distal to the ligament of Treitz. The jejunal loop is brought up antecolic and antegastric and is anastomosed to the pouch to form the gastrojejunostomy with a linear stapler and a running barbed wire. The Peterson-window is closed with staples in patients in the Spaarne Gasthuis [17, 18]. The OAGB is shown in Fig. 3.

### Outcome measures

The primary outcome measure of the DIABAR-trial is glycemic control, as measured by the difference in Hba1c 12 months after RYGB and OAGB. Glycemic control at 6 months, 2–5 and 10 years of follow-up, and the remission of T2DM at 1–5 and 10 years of follow-up are secondary outcome measures. For remission of diabetes, we will use the proposed definition according to ADA consensus report 2021 primarily (Hba1c < 6.5%), and in a secondary analysis, the more strict cut-off < 5.7% will be used [19]. Other secondary outcome measures are related to the interplay of obesity, NAFLD/NASH, and cardio-vascular performance. Table 1 shows the schedule of enrolment and study procedures (SPIRIT-format). All measurements will be performed by the researchers and physicians according to a standard operating procedure.

*Demographics* consist of age, sex, ethnic background, medical history, use of medication, history of weight-change, intoxications, educational level, employment status, and physical activity. Prior to surgery, patients will be asked about their expectations of metabolic surgery in terms of weight loss.

*Biometric data* consist of weight, height, waist to hip ratio, temperature, bioelectrical impedance (BIA), electrocardiogram (ECG), blood pressure, pulse, stroke volume, cardiac output, and systemic vascular resistance (Nexfin).

*Dutch versions of validated questionnaires* are used for evaluation of psychological measures. This includes quality of life, self-efficacy, hunger and craving, social support, depression, and body image, as previously described in the protocol of the BARIA-study [15]. Food intake is registered using food diaries. Gastro-intestinal complaints are assessed with the gastro-intestinal quality of life (GIQLI) questionnaire [20].

*Peripheral blood* will be drawn at various time points as part of the regular follow-up and includes hemoglobin, leukocytes, C-reactive protein, HbA1c, glucose, c-peptide (only baseline), electrolytes, kidney function, hepatic enzymes, lipid profile, iron, vitamins, and thyroid profile.

*Mixed meal test*, a 2-h oral mixed meal test (MMT), will be performed three times in the course of the study [21]. During the MMT, the patient will consume two 125 ml Nutridrink compact drinks (Nutricia®). At set time points during this test, blood will be obtained through an intravenous line and will be used to measure insulin sensitivity, plasma metabolites, and bile acids.

*Oral swabs and fecal and urine samples* will be collected at various time points. This includes 24-h and morning fecal and urine samples, as well as a gingival swab.

*Ultrasound of the gallbladder* will be performed to detect gallstones prior to and during follow-up after surgery.

*Surgery*: patients will either undergo a RYGB or OAGB according to randomization. During surgery, the following tissue biopsies will be obtained: subcutaneous fat from any of the trocar incisions, tissue from the greater omentum and visceral fat from the omental appendices of the colon transversum, and jejunum at the jejuno-jejunostomy site (only in patients that undergo a RYGB) and liver biopsy (of segment 3 or 5). In selected cases, and only if considered safe by the surgeon, blood will be drawn from the portal vein at the beginning of the surgery.

*Surgical endpoints* are time from incision of the skin to closure of the wound, conversion, hospital stay, readmission, and complications and mortality ≤ 30 days and > 30 days of surgery.

*Re-operation/endoscopys*: in case a patient enrolled from the Spaarne Gasthuis will undergo another, and non-acute, abdominal surgery longer than 30 days after the RYGB or OAGB, the patient will be asked for informed consent to obtain biopsies from liver and fat depots. In case of cholecystectomy, the patient will be asked for consent to collect bile acid and gallbladder tissue. If a patient enrolled from the Spaarne Gasthuis needs to undergo an endoscopy indicated by upper gastro-intestinal complaints, the patient will be asked for permission to obtain a biopsy of the small intestine.

### Data handling and storage

All electronic data will be stored in an electronic case record form, which was designed in the program Castor Electronic Data Capture (Ciwit BV, the Netherlands). This software is compliant with “good clinical practice.” All data will be handled confidentially and will be registered under a unique code in accordance with the Dutch Personal Data Protection Act (Wbp).

Study materials will be stored for a period of 20 years at − 80° Celsius in the DIABAR-biobank. Blood samples and other materials collected during the trial will be subjected to extensive testing, as further elaborated in the study-protocol of the closely related BARIA-study [15]. Briefly, this testing encompasses evaluation of plasma metabolites in peripheral blood samples taken prior to and after the MMT and in portal vein blood; assessment of gut microbiome and metabolites from fecal samples; qPCR of liver, jejunal, and fat tissue biopsies; assessment of intestinal immunological cells in Peyer’s patches, in adipose and liver tissue and in peripheral blood samples; and evaluation of immunological parameters in small intestinal and adipose tissue.

### Statistical analysis

#### Power calculation and sample size

Sample size calculation was performed based on a retrospective analysis of patients after RYGB (*n* = 90) and OAGB (*n* = 37). Patients were matched in a 3:1 ratio on baseline age, BMI, and gender. The mean pre-operative Hba1c in the RYGB group was 7.5 ± 1.4% and 7.5 ± 1.5% in the OAGB group (*p* = 0.97). Null hypothesis is as follows: there is no difference in decrease of Hba1c at 12 months after RYGB or OAGB in patients with T2DM. Alternative hypothesis: the decrease in Hba1c after OAGB is higher than after RYGB in patients with T2DM at 12 months after surgery. For sample size calculation, the mean Hba1c of both groups was compared with a two-sided significance level of *P* < 0.05 and a power of 80%. Based on the retrospective results, 100 subjects per group will be required. Considering a 10% dropout rate, 220 patients are needed.

#### Analysis of endpoints

The statistical analyses will be performed using SPSS statistical software (IBM corporation, New York, USA). Data will be expressed as mean, standard deviation, or median and range, as appropriate. Differences in categorical variables will be evaluated with the chi-square or the Fisher exact test, as appropriate; continuous data will be compared with the Student *t* test or Mann-Whitney *U*. Multivariate analysis and ANOVA for repeated measures will be used. *P*-values < 0.05 will be considered statistically significant. Clinical complications will be evaluated by an independent adjudication committee. An interim analysis will be performed at 60 patients per group. A modified intention to treat analysis will be performed (all randomized patients that initiate treatment in the arm they are allocated to, i.e., exempting those who do not undergo surgery at all) in which “last value carried forward” will be used for missing observations. A per-protocol analysis will be carried out as well to account for effects of revisional surgery (i.e., revision from OAGB to RYGB). For this analysis, only data of patients gathered before revisional surgery will be used.

### Ethics and permissions

The study will be conducted in accordance with the Declaration of Helsinki and “good clinical practice.” Ethical approval has initially been granted by the Institutional Review Board of Slotervaartziekenhuis en Reade, Amsterdam, the Netherlands. In 2019, the study was transferred to and approved by the Institutional Review board of Amsterdam UMC, location AMC, Amsterdam, the Netherlands (registered under NL61882.048.17). A data monitoring committee has not been installed. Auditing will be performed (at least) annually, independent from investigators and the sponsor.

### SPIRIT checklist

The SPIRIT checklist has been added as a supplementary file [22]. In Fig. 1 and in Table 1 study enrolment and study procedures are shown, according to the SPIRIT format.

### Trial status

The first patient was enrolled and randomized on October 23, 2017. The study is ongoing. At the time of writing, 86 patients are enrolled in the Spaarne Gasthuis and 14 in the Franciscus Gasthuis. Recruitment is estimated to be completed in 2024. Current trial protocol is version 8.1, July 2020.

Results of the trial will be presented in a manuscript and offered to a peer-reviewed journal.

## Discussion

The STAMPEDE-trial has shown that surgery is more effective than medical treatment in patients with obesity and T2DM [2]. Still, it has not been established which metabolic surgery is the most powerful in the treatment of T2DM. In the Netherlands, the RYGB is the most performed bariatric and metabolic procedure [23]. With a peri-operative mortality rate between 0.1 and 0.41%, a rate of serious adverse events within the first 30 days after surgery ranging from 1.38 to 8.3%, and sustainable weight loss at follow-up, the RYGB can be considered a safe treatment for severe obesity [24–30]. The remission of T2DM after RYGB is reported to be between 50 and 80% depending on length of follow-up [4, 5, 31–33]. Although the RYGB has good results, it is considered an advanced and complex laparoscopic procedure, with a learning curve between 50 and 100 cases [34]. The OAGB was introduced as a simplified version of the RYGB and from start the OAGB has reported promising results in terms of weight loss, post-operative morbidity and mortality [35–43]. In single-arms studies, the short-term remission of T2DM is reported to be between 84 and 92% [42, 43]. The DIABAR-trial will demonstrate which of these two procedures is the most beneficial in patients suffering from T2DM and severe obesity. This information will aid the patient and physician in selecting the most potent surgical treatment. By extension, it is important to weigh the metabolic advantages of these procedures against the concerns in the post-operative course. Besides the more obvious surgical complications, such as post-operative bleeding and anastomotic leakage, seen after both RYGB and OAGB, there are some procedure-specific concerns. Internal herniation (IH) is a potentially serious complication after RYGB as it harbors the risk of small bowel obstruction and subsequent small bowel ischemia; after OAGB, IH is seldomly seen [44–47]. The OAGB has been surrounded by controversy for its potential to cause biliary reflux and to induce subsequent metaplasia, dysplasia, and carcinoma of the gastric remnant and/or the esophagus [48, 49].

The underlying physiological and molecular mechanisms responsible for the antidiabetic effects of metabolic surgery have not been fully understood. Proposed theories include acute reduced caloric intake possibly-in part-responsible for the weight-independent antidiabetic effect occurring directly post-surgery, as well as a middle-to long-term antidiabetic effect of surgery-induced weight loss [50]. Rapidly occurring enhanced glycemic control might also be due to altered anatomy and signaling/sensing of the gastro-intestinal tract, formulated in the foregut and hindgut hypothesis [51, 52]. Among other factors that are recognized to be of potential influence are the immune system, the intestinal microbiome, and altered bile acid metabolism [53–56]. Also, there is an association between obesity, non-alcoholic fatty liver disease (NALFD), and insulin resistance/T2DM, with reports of the presence of (some form of) NAFLD in up to 80% of patients undergoing weight loss surgery [57, 58]. It is likely that not one (dominant) factor or one mechanism is responsible for the beneficial glucoregulatory effects of metabolic surgery but that the effects are due to an interplay of various pathways. The DIABAR-trial grants the unique opportunity to assess extensive data of patients with T2DM and obesity after two different metabolic procedures and to explore driving mechanisms in metabolic response.

A few limitations have to be acknowledged. First, for logistic reasons, patients will not be stratified upon the use of insulin or the duration of T2DM. Second, the authors are aware of and responsible for the extent of the study procedures patients are subjected to. Therefore, the study is performed by a research nurse, who stays in close contact with participating patients and monitors how patients perceive their participation in the study. To meet with the patient’s time investment, patients receive a financial reimbursement. Third, patients are required to lose weight prior to surgery. This might influence the metabolic changes induced by surgery; yet patients in both arms of the study are projected to the same pre-surgical weight loss and it lowers the surgical risk.

## Conclusion

The DIABAR-trial is the first randomized controlled trial to evaluate the glycemic response after RYGB and OAGB in a severe obese population diagnosed with T2DM. This will provide patients and physicians with the information needed to come to an evidence based decision upon the most potent metabolic surgery. The DIABAR-trial will also generate a large dataset which will be used to phenotype subjects with T2DM prior to and after surgery. This data will contribute to a deeper understanding of the mechanisms that drive the remission of T2DM in this population by identification of microbial, immunological and metabolic markers for metabolic response.

## Supplementary Information

**Additional file 1.**

## Data Availability

Reasonable requests for access to the full protocol can be made to the corresponding author.

## Abbreviations

ASA: American Society of Anesthesiologist Classification
AUMC: Amsterdam University Medical Center
BIA: Bioelectrical impedance analysis
BMI: Body mass index
MMT: Mixed meal test
OAGB: One-anastomosis gastric bypass
RYGB: Roux-en-Y gastric bypass
NAFLD: Non-alcoholic fatty liver disease
NASH: Non-alcoholic steatohepatitis
T2DM: Type 2 diabetes mellitus
