# Supplementary material for: The effects of laparoscopic Roux-en-Y gastric bypass and one-anastomosis gastric bypass on glycemic control and remission of type 2 diabetes mellitus: study protocol for a multi-center randomized controlled trial (the DIABAR-trial)
Source: Trials. 2022 Oct 22;23:900. doi: 10.1186/s13063-022-06762-3 (PMC9588204; doi:10.1186/s13063-022-06762-3)
Supplement: Supplementary file 1 — Additional file 1. [file 13063_2022_6762_MOESM1_ESM.docx]

# **SPIRIT Checklist for *Trials***

Complete this checklist by entering the page and line numbers where each of the items listed below can be found in your manuscript.

Your manuscript may not currently address all the items on the checklist. Please modify your text to include the missing information. If you are certain that an item does not apply, please state "n/a" and provide a short explanation. **Leaving an item blank or stating “n/a” without an explanation will lead to your manuscript being returned before review.**

Upload your completed checklist as an additional file when you submit to *Trials*. You must reference this additional file in the main text of your protocol submission. The completed SPIRIT figure must be included within the main body of the protocol text and can be downloaded here: [http://www.spirit-statement.org/schedule-of-enrolment-interventions-and-assessments/](about:blank)

In your methods section, please state that you used the SPIRIT reporting guidelines, and cite them as:

Chan A-W, Tetzlaff JM, Gøtzsche PC, Altman DG, Mann H, Berlin J, Dickersin K, Hróbjartsson A, Schulz KF, Parulekar WR, Krleža-Jerić K, Laupacis A, Moher D. SPIRIT 2013 Explanation and Elaboration: Guidance for protocols of clinical trials. BMJ. 2013;346:e7586

|  |  | **Reporting Item** | **Page and Line Number** | **Reason if not applicable** |
| --- | --- | --- | --- | --- |
| **Administrative information** | | | | |
| Title | [#1](about:blank#1) | Descriptive title identifying the study design, population, interventions, and, if applicable, trial acronym | Page 1, line 1-3 |  |
| Trial registration | [#2a](about:blank#2a) | Trial identifier and registry name. If not yet registered, name of intended registry | Page 2, line 73-74 |  |
| Trial registration: data set | [#2b](about:blank#2b) | All items from the World Health Organization Trial Registration Data Set | Page 2, line 73-74 |  |
| Protocol version | [#3](about:blank#3) | Date and version identifier | Page 11, line 288 |  |
| Funding | [#4](about:blank#4) | Sources and types of financial, material, and other support | Page 14, line 381-389 |  |
| Roles and responsibilities: contributorship | [#5a](about:blank#5a) | Names, affiliations, and roles of protocol contributors | Page 15, line 391-400 |  |
| Roles and responsibilities: sponsor contact information | [#5b](about:blank#5b) | Name and contact information for the trial sponsor | Page 15, line 391 |  |
| Roles and responsibilities: sponsor and funder | [#5c](about:blank#5c) | Role of study sponsor and funders, if any, in study design; collection, management, analysis, and interpretation of data; writing of the report; and the decision to submit the report for publication, including whether they will have ultimate authority over any of these activities | Page 15, line 391-400 |  |
| Roles and responsibilities: committees | [#5d](about:blank#5d) | Composition, roles, and responsibilities of the coordinating centre, steering committee, endpoint adjudication committee, data management team, and other individuals or groups overseeing the trial, if applicable (see Item 21a for data monitoring committee) | Page 10, line 267-268 |  |
| **Introduction** |  |  | Page 3,line 77 - 96 |  |
| Background and rationale | [#6a](about:blank#6a) | Description of research question and justification for undertaking the trial, including summary of relevant studies (published and unpublished) examining benefits and harms for each intervention | Page 3,line 77 - 96 |  |
| Background and rationale: choice of comparators | [#6b](about:blank#6b) | Explanation for choice of comparators | Page 3,line 82 - 91 |  |
| Objectives | [#7](about:blank#7) | Specific objectives or hypotheses | Page 3, line 91 - 96 |  |
| Trial design | [#8](about:blank#8) | Description of trial design including type of trial (eg, parallel group, crossover, factorial, single group), allocation ratio, and framework (eg, superiority, equivalence, non-inferiority, exploratory) | Page 3, line 88-89 |  |
| **Methods: Participants, interventions, and outcomes** | | | | |
| Study setting | [#9](about:blank#9) | Description of study settings (eg, community clinic, academic hospital) and list of countries where data will be collected. Reference to where list of study sites can be obtained | Page 4 line 111-122 |  |
| Eligibility criteria | [#10](about:blank#10) | Inclusion and exclusion criteria for participants. If applicable, eligibility criteria for study centres and individuals who will perform the interventions (eg, surgeons, psychotherapists) | Page 5 and 6, line 133-157 |  |
| Interventions: description | [#11a](about:blank#11a) | Interventions for each group with sufficient detail to allow replication, including how and when they will be administered | Page 6 and 7, line 169- 190 |  |
| Interventions: modifications | [#11b](about:blank#11b) | Criteria for discontinuing or modifying allocated interventions for a given trial participant (eg, drug dose change in response to harms, participant request, or improving / worsening disease) | n/a | Participants undergo the surgical intervention, RYGB or OAGB as allocated.  Patients can withdraw from the study at any time. Patients with missing data on the primary endpoint will be considered a drop-out. If patients withdraw from the study after allocation, but before surgery, they will be considered a drop-out. Patients that receive revisional surgery are considered a drop-out from the time of revision. However, data gathered before revision will be used up to the point at which the data is no longer available. |
| Interventions: adherance | [#11c](about:blank#11c) | Strategies to improve adherence to intervention protocols, and any procedures for monitoring adherence (eg, drug tablet return; laboratory tests) | Page 12 , line 330-332 | Participants undergo the surgical intervention, RYGB or OAGB as allocated; there is no further intervention to adhere to after surgery is performed. Our study nurse keeps in touch with the patients during follow-up and monitors how the study is perceived |
| Interventions: concomitant care | [#11d](about:blank#11d) | Relevant concomitant care and interventions that are permitted or prohibited during the trial | n/a | The implementation of RYGB or OAGB does not require any alterations in usual care pathways. This includes use of any medication. There are no restrictions regarding concomitant care during the trial. |
| Outcomes | [#12](about:blank#12) | Primary, secondary, and other outcomes, including the specific measurement variable (eg, systolic blood pressure), analysis metric (eg, change from baseline, final value, time to event), method of aggregation (eg, median, proportion), and time point for each outcome. Explanation of the clinical relevance of chosen efficacy and harm outcomes is strongly recommended | Page 7 and 8, line 191-236 |  |
| Participant timeline | [#13](about:blank#13) | Time schedule of enrolment, interventions (including any run-ins and washouts), assessments, and visits for participants. A schematic diagram is highly recommended (see Figure) | Table 1 and Figure 2 grant an overview of study flow and study visits, with additional description on Page 7 and 8, line 188-233 |  |
| Sample size | [#14](about:blank#14) | Estimated number of participants needed to achieve study objectives and how it was determined, including clinical and statistical assumptions supporting any sample size calculations | Page 9, line 248-257 |  |
| Recruitment | [#15](about:blank#15) | Strategies for achieving adequate participant enrolment to reach target sample size | Page 6, line 158-163 |  |
| **Methods: Assignment of interventions (for controlled trials)** | | | | |
| Allocation: sequence generation | [#16a](about:blank#16a) | Method of generating the allocation sequence (eg, computer-generated random numbers), and list of any factors for stratification. To reduce predictability of a random sequence, details of any planned restriction (eg, blocking) should be provided in a separate document that is unavailable to those who enrol participants or assign interventions | Page 6, line 163-168 |  |
| Allocation concealment mechanism | [#16b](about:blank#16b) | Mechanism of implementing the allocation sequence (eg, central telephone; sequentially numbered, opaque, sealed envelopes), describing any steps to conceal the sequence until interventions are assigned | Page 6, line 163-166 |  |
| Allocation: implementation | [#16c](about:blank#16c) | Who will generate the allocation sequence, who will enrol participants, and who will assign participants to interventions | Page 6, line 163-166 |  |
| Blinding (masking) | [#17a](about:blank#17a) | Who will be blinded after assignment to interventions (eg, trial participants, care providers, outcome assessors, data analysts), and how | page 4, line 112 | The DIABAR-trial is an open randomized controlled trial, |
| Blinding (masking): emergency unblinding | [#17b](about:blank#17b) | If blinded, circumstances under which unblinding is permissible, and procedure for revealing a participant’s allocated intervention during the trial | Page 4, line 112 | Given the open label design of the study, unblinding will not occur. |
| **Methods: Data collection, management, and analysis** | | | | |
| Data collection plan | [#18a](about:blank#18a) | Plans for assessment and collection of outcome, baseline, and other trial data, including any related processes to promote data quality (eg, duplicate measurements, training of assessors) and a description of study instruments (eg, questionnaires, laboratory tests) along with their reliability and validity, if known. Reference to where data collection forms can be found, if not in the protocol | Page 4 line 119-122, Page 7 and 8, line 191-236,Page 9 line 242-249 |  |
| Data collection plan: retention | [#18b](about:blank#18b) | Plans to promote participant retention and complete follow-up, including list of any outcome data to be collected for participants who discontinue or deviate from intervention protocols | Page 10, line 268-273 |  |
| Data management | [#19](about:blank#19) | Plans for data entry, coding, security, and storage, including any related processes to promote data quality (eg, double data entry; range checks for data values). Reference to where details of data management procedures can be found, if not in the protocol | Page 4 line 119-122 and Page 9 line 238 - 249 |  |
| Statistics: outcomes | [#20a](about:blank#20a) | Statistical methods for analysing primary and secondary outcomes. Reference to where other details of the statistical analysis plan can be found, if not in the protocol | Page 10, line 261 - 273 |  |
| Statistics: additional analyses | [#20b](about:blank#20b) | Methods for any additional analyses (eg, subgroup and adjusted analyses) | Page 10, line 261 - 273 |  |
| Statistics: analysis population and missing data | [#20c](about:blank#20c) | Definition of analysis population relating to protocol non-adherence (eg, as randomised analysis), and any statistical methods to handle missing data (eg, multiple imputation) | Page 10, line 261 - 273 |  |
| **Methods: Monitoring** | | | | |
| Data monitoring: formal committee | [#21a](about:blank#21a) | Composition of data monitoring committee (DMC); summary of its role and reporting structure; statement of whether it is independent from the sponsor and competing interests; and reference to where further details about its charter can be found, if not in the protocol. Alternatively, an explanation of why a DMC is not needed | Page 10, line 279-281 | There is no DMC installed for the DIABAR trial, as RYGB has been standard care over years. The OAGB is derived from the RYGB, and therefore has been perceived as good to incorporate in standard care. In addition, before the study OAGB was already performed in the participating centers in usual care. |
| Data monitoring: interim analysis | [#21b](about:blank#21b) | Description of any interim analyses and stopping guidelines, including who will have access to these interim results and make the final decision to terminate the trial | Page 10, line 268and further elaborated in the study prococol | Further elaboration in the study protocol   ‘*The investigator/sponsor will notify the accredited METC of the end of the study within a period of 8 weeks. The end of the study is defined as the last patient’s last visit.  The sponsor will notify the METC immediately of a temporary halt of the study, including the reason of such an action.  In case the study is ended prematurely, the sponsor will notify the accredited METC within 15 days, including the reasons for the premature termination..’* |
| Harms | [#22](about:blank#22) | Plans for collecting, assessing, reporting, and managing solicited and spontaneously reported adverse events and other unintended effects of trial interventions or trial conduct | n/a | This has been elaborated in the study protocol, not in the manuscript:  *‘6.2.1 Adverse events (AEs)*  *Adverse events are defined as any undesirable experience occurring to a subject during the study, whether or not considered related to the trial procedure. All adverse events reported spontaneously by the subject or observed by the investiga¬tor or his staff will be recorded.*  *6.2.2 Serious adverse events (SAEs)*  *A SAE is any untoward medical occurrence or effect that:*  *- results in death;  - is life threatening (at the time of the event);  - requires hospitalisation or prolongation of existing inpatients’ hospitalisation;*  *- results in persistent or significant disability or incapacity; - is a congenital anomaly or birth defect;  - or any other important medical event that did not result in any of the outcomes listed above due to medical or surgical intervention but could have been based upon appropriate judgement by the investigator. An elective hospital admission will not be considered as a SAE.*  *Only SAEs that can be directly linked to the procedure will be reported through the web portal ToetsingOnline to the accredited METC that approved the protocol, within 15 days after the sponsor has first knowledge of the serious adverse reactions. All other SAEs will be recorded, and will be reported as line listing through the web portal Toetsingonline.*  *SAEs that result in death or are life threatening should be reported expedited. The expedited reporting will occur not later than 7 days after the responsible investigator has first knowledge of the adverse reaction. This is for a preliminary report with another 8 days for completion of the report’* |
| Auditing | [#23](about:blank#23) | Frequency and procedures for auditing trial conduct, if any, and whether the process will be independent from investigators and the sponsor | Page 10, line 279-281 | A DMC has not been installed, (item21a). In addition to daily communication between coordinating researcher and other researchers and the trial nurse, the trial group (Principal investigators, coordinating researchers, researchers and trial nurse) meet at least twice a year to review the study and study processes. Auditing will be performed unannounced, independently from investigators and the sponsor, at least once a year. |
| **Ethics and dissemination** | | | | |
| Research ethics approval | [#24](about:blank#24) | Plans for seeking research ethics committee / institutional review board (REC / IRB) approval | Page 10, line 274-281 |  |
| Protocol amendments | [#25](about:blank#25) | Plans for communicating important protocol modifications (eg, changes to eligibility criteria, outcomes, analyses) to relevant parties (eg, investigators, REC / IRBs, trial participants, trial registries, journals, regulators) | n/a | All proposed substantial changes will be communicated with the sponsor, funder and the PI. The revised protocol will be sent to the participating centers and after internal review, the amendment will be proposed to the institutional review board for approval. After approval, the protocol amendment and the updated protocol will be added to the investigator site file. Non-substantial amendments will not be notified to the institutional review board. They will be recorded and filed by the sponsor. Any deviations from the protocol will be fully documented in a breach report form. The clinical trial registry will be updated as appropriate. |
| Consent or assent | [#26a](about:blank#26a) | Who will obtain informed consent or assent from potential trial participants or authorised surrogates, and how (see Item 32) | Page 6, line 162-168 |  |
| Consent or assent: ancillary studies | [#26b](about:blank#26b) | Additional consent provisions for collection and use of participant data and biological specimens in ancillary studies, if applicable | Page 8 and 9, line 231-232 |  |
| Confidentiality | [#27](about:blank#27) | How personal information about potential and enrolled participants will be collected, shared, and maintained in order to protect confidentiality before, during, and after the trial | Page 9, line 238-241 |  |
| Declaration of interests | [#28](about:blank#28) | Financial and other competing interests for principal investigators for the overall trial and each study site | Page 14 and 15, line 375-3389 |  |
| Data access | [#29](about:blank#29) | Statement of who will have access to the final trial dataset, and disclosure of contractual agreements that limit such access for investigators | Page 10, line 264-265 |  |
| Ancillary and post trial care | [#30](about:blank#30) | Provisions, if any, for ancillary and post-trial care, and for compensation to those who suffer harm from trial participation | n/a | Elaborated in the study protocol, not the manuscript:  *‘The sponsor/investigator has a liability insurance which is in accordance with article 7, subsection 6 of the WMO.*  *The sponsor (also) has an insurance which is in accordance with the legal requirements in the Netherlands (Article 7 WMO and the Measure regarding Compulsory Insurance for Clinical Research in Humans of 1ste July 2015). This insurance provides cover for damage to research subjects through injury or death caused by the study.’* |
| Dissemination policy: trial results | [#31a](about:blank#31a) | Plans for investigators and sponsor to communicate trial results to participants, healthcare professionals, the public, and other relevant groups (eg, via publication, reporting in results databases, or other data sharing arrangements), including any publication restrictions | Page 11, line 289 | After completion of the DIABAR-trial and final analyses, the results will be presented in a manuscript and offered to a peer-reviewed journal. At due time, when the manuscript is accepted, social media might be used to further disseminate the findings. Data will also be presented at (inter)national congresses, if accepted for presentation. |
| Dissemination policy: authorship | [#31b](about:blank#31b) | Authorship eligibility guidelines and any intended use of professional writers | n/a | Currently there is no intention to use professional writers. |
| Dissemination policy: reproducible research | [#31c](about:blank#31c) | Plans, if any, for granting public access to the full protocol, participant-level dataset, and statistical code | Page 15, line 404 | The full protocol is available from the corresponding author on reasonable request.  Reasonable requests for access to the full dataset and statistical code can be made to the corresponding author and will be reviewed by the PIs and sponsor. |
| **Appendices** | | | | |
| Informed consent materials | [#32](about:blank#32) | Model consent form and other related documentation given to participants and authorised surrogates | n/a | All patients receive patient oral and written information on the study at the outpatient clinic, which includes an informed consent form for participation in the study and storing of study materials. This documentation has all been approved by the institutional review board.   The patient information, as well as the informed consentform are available in Dutch, not in English. A clean-copy of the patient information and the informed consent form are available from the corresponding author on request. |
| Biological specimens | [#33](about:blank#33) | Plans for collection, laboratory evaluation, and storage of biological specimens for genetic or molecular analysis in the current trial and for future use in ancillary studies, if applicable | Page 9 line 242-249 |  |

It is strongly recommended that this checklist be read in conjunction with the SPIRIT 2013 Explanation & Elaboration for important clarification on the items. Amendments to the protocol should be tracked and dated. The SPIRIT checklist is copyrighted by the SPIRIT Group under the Creative Commons “[Attribution-NonCommercial-NoDerivs 3.0 Unported](about:blank)” license. This checklist can be completed online using https://www.goodreports.org/, a tool made by the EQUATOR Network in collaboration with Penelope.ai
